# A Cumulative Risk Perspective for Occupational Health and Safety (OHS) Professionals

**DOI:** 10.3390/ijerph17176342

**Published:** 2020-08-31

**Authors:** Richard Todd Niemeier, Pamela R.D. Williams, Alan Rossner, Jane E. Clougherty, Glenn E. Rice

**Affiliations:** 1 Division of Science Integration, National Institute for Occupational Safety and Health, Cincinnati, OH 45226, USA; 2 E Risk Sciences, LLP, Lafayette, CO 80026, USA; pwilliams@erisksciences.com; 3 Institute for a Sustainable Environment, Clarkson University, Potsdam, NY 13699, USA; arossner@clarkson.edu; 4 Department of Environmental and Occupational Health, Dornsife School of Public Health, Drexel University, Philadelphia, PA 19104, USA; jec373@drexel.edu; 5 Center for Public Health and Environmental Assessment, Office of Research and Development, USA EPA, Cincinnati, OH 45268, USA; rice.glenn@epa.gov

**Keywords:** CRA, cumulative risk, exposure assessment, industrial hygiene, workplace, OHS professionals

## Abstract

Cumulative risk assessment (CRA) addresses the combined risk associated with chemical and non-chemical exposures. Although CRA approaches are utilized in environmental and ecological contexts, they are rarely applied in workplaces. In this perspectives article, we strive to raise awareness among occupational health and safety (OHS) professionals and foster the greater adoption of a CRA perspective in practice. Specifically, we provide an overview of CRA literature as well as preliminary guidance on when to consider a CRA approach in occupational settings and how to establish reasonable boundaries. Examples of possible workplace co-exposures and voluntary risk management actions are discussed. We also highlight important implications for workplace CRA research and practice. In particular, future needs include simple tools for identifying combinations of chemical and non-chemical exposures, uniform risk management guidelines, and risk communication materials. Further development of practical CRA methods and tools are essential to meet the needs of complex and changing work environments.

## 1. Introduction 

Occupational health and safety (OHS) professionals routinely assess many different chemical and non-chemical (e.g., biologic, radiologic, physical, psychological) exposures in the workplace [1,2]. These are commonly referred to as “stressors” in the industrial hygiene and environmental risk assessment literature [1,3]. In occupational settings, each identified stressor is typically evaluated independently by comparing the exposure concentration to an occupational exposure limit (OEL) or other health-based guidelines. Formally, evaluating the combined risk from co-exposure to multiple stressors in occupational (and non-occupational) domains is not common practice [4,5,6]. Lifestyle or personal risk factors that can modify the effect of chemical or non-chemical exposures in the workplace, such as age, obesity, smoking, alcohol consumption, drug use, or nutritional and health status, are likewise not usually considered in occupational health risk assessments [7]. Guidance is needed on reliable and effective strategies to identify, evaluate, and manage such complex scenarios in occupational settings. 

Cumulative risk assessment (CRA) is an approach designed to address similar types of concerns in environmental and ecological contexts [3,8,9,10]. CRA is defined as “an analysis, characterization, and possible quantification of the combined risks to health or the environment from multiple agents or stressors” [8]. CRA incorporates and provides additional tools for implementing the traditional 4-step risk assessment paradigm that comprises hazard identification, dose–response assessment, exposure assessment, and risk characterization [11,12]. There are at least two important distinctions when evaluating cumulative risks. First, is the requirement that at least two stressors (chemical or non-chemical) from all routes of exposure that affect a common health outcome (via the same or different biological mechanism or mode of action) be included in the assessment. Second, is that the combined health risk associated with the co-exposures must be evaluated. CRAs are also geared towards a population-based approach in which the primary stressors affecting a particular group are examined in the context of their combined exposures and effects on public health, and potential vulnerabilities and susceptibilities in the population are emphasized [3]. CRAs therefore incorporate the concept of aggregate exposure (i.e., exposure to same stressor from multiple exposure routes) but differ from cumulative exposure (i.e., co-exposure to multiple stressors that may have different health effects). Because many terms used in CRAs or similar approaches have not always been well-defined or consistently used [13], we attempt to clarify some of these terms (Table 1). 

A number of review articles provide an overview of the CRA literature and limited applications [4,5,9,10,27,28,29,30,31,32,33,34]. Overall, this novel approach has been touted as essential for informing decision making and risk management because it has the potential to provide a broader and more comprehensive understanding of multi-exposure effects in a population [12,35]. However, only a few specific models or frameworks have been proposed for conducting CRAs in practice [3,8,29,36,37,38,39]. CRAs conducted thus far also have been criticized for being limited in scope and failing to incorporate non-chemical stressors, personal risk factors, and population vulnerabilities or susceptibilities [12,20]. Additionally, past CRA applications have focused primarily on environmental or community settings, with minimal consideration of occupational exposures and their contribution to total health risk [4,5,6]. One exception is the related “Total Worker Health” (TWH) program, which acknowledges risk factors related to work (e.g., stress, poor ergonomics) that contribute to health problems previously considered unrelated to work [40]. Although examples of combined exposures in the occupational environment have been studied [41,42,43,44,45], the authors are aware of no occupationally based CRAs that have been conducted or published to date. 

Because explicit guidance is not yet available for conducting comprehensive or quantitative CRAs within occupational settings, few OHS professionals are familiar with or willing to adopt this approach. Real-world case study examples and practical applications are also lacking. However, despite these challenges, several components of CRA’s still can be implemented in the workplace today as an interim step. Efforts to introduce CRA concepts and techniques in the workplace began about 10 years ago with some continued presentations to the OHS community over time [46,47]. We believe it is necessary to move beyond discussions of higher-level concepts to more tangible applications in the workplace.

Here, we strive to raise further awareness and understanding of CRA approaches among OHS professionals worldwide and promote the greater adoption of a CRA perspectives when evaluating health and safety risks in the workplace. Our specific objectives are three-fold: (1) provide preliminary guidance for determining when a CRA approach may be beneficial in occupational settings and establishing realistic boundaries for analysis, (2) present several hypothetical examples of co-exposures in the workplace that could lead to combined health risks and risk management actions that can be taken in these situations, and (3) discuss important implications for occupationally based CRA research and practice in the future. Utilizing a CRA approach can help improve the reliability of and confidence in occupational health assessments and risk management decisions by focusing more holistically on how multiple exposures can interact to contribute to a health outcome. Taking a CRA perspective in occupational settings can also enable the profession to better meet the needs of complex and changing work environments. Although important considerations, this paper does not address broader social or policy issues, such as employee and employer responsibilities, regulatory jurisdictional issues, or personal privacy issues. These complex issues will take time to carefully examine and will be necessary if CRA approaches are to be developed more broadly or adopted within regulatory contexts, but they do not need to be resolved before preliminary steps can be taken to evaluate worker health from a CRA perspective. 

## 2. When to Consider a CRA Approach in Occupational Settings

Based on our review of the available literature and professional experiences, we provide preliminary guidance on when to consider using a CRA approach in occupational settings and how to establish realistic boundaries. We offer an initial (simple and practical) framework for OHS professionals to follow, recognizing that a more comprehensive framework should be developed in the future as more information becomes available with respect to identifying co-exposures of concern and toxicological interactions among stressors. 

### 2.1. Boundaries and Scale of Occupationally Based CRAs 

Similar types of chemical and non-chemical exposures and personal risk factors are present in both occupational and non-occupational domains (Table 2). Although a complete evaluation of all relevant exposures and risk factors in both domains would be ideal when conducting a health risk assessment, this is generally not feasible or practical for real-world settings. Recognizing that the boundaries and scale of any CRA must be manageable and provide relevant information for practical decision-making, the National Research Council [12] advises that CRAs be tailored to address only those exposures or risk factors for which one or more risk management options apply. Moretto, Moretto [37] and Solomon, Wilks [48] also recommend an upfront “gatekeeper” step for determining whether a CRA is necessary in which for any and all chemical exposures included in a CRA: (1) there must be sufficient evidence for both co-exposure and common toxicity and (2) the exposure levels must warrant at least minimal concern individually, but not be so high as to require immediate intervention. We note that individual exposures below levels associated with toxicity can still be of concern when in combination with similar exposures.

In keeping with this prior guidance, we recommend that occupationally based CRAs focus initially only on those exposures that are within the purview of the OHS practitioner and under an employer’s control (i.e., within the fence line). Related exposures and personal risk factors that are not generally within the purview of the OHS practitioner and are outside an employer’s control (i.e., outside the fence line) could be considered if they meet one or more of the following criteria:

(a) are the same type of exposure and substantially add to the workplace exposure (e.g., noise exposure from target shooting as a hobby plus occupational noise exposure);
(b) act on the same biological pathways (e.g., are anticipated to be dose additive with the exposure occurring within the fence line, such as exposure to dioxin-like compounds at work) or cause the same health effect (e.g., this could include exposures that potentially act through response addition, among other possibilities);
(c) may plausibly modify (i.e., alter or change) the health effect of concern or substantially alter population vulnerabilities or susceptibilities.

Once all relevant exposures and personal risk factors have been identified, they can be evaluated together in a quantitative or qualitative manner following a standardized sequence of steps. The steps should move consistently towards building an integrated assessment of the total burden of a given set of exposures on worker health. One recommended model for incorporating non-chemical exposures into CRAs has been presented by Clougherty and Levy [39]. 

### 2.2. Initiating Factors Prompting CRAs in the Workplace 

It is useful to consider which scenarios or criteria might trigger the need for a CRA approach in the workplace. These are likely to be similar to the types of “initiating factors” identified for environmental settings [3]. The presence of multiple exposures in the workplace, particularly if non-negligible exposures are measured, should prompt OHS professionals to consider a CRA perspective using a stressor (exposure)-based approach [49] Under this approach, the first step is to identify all chemical and non-chemical exposures as well as the specific group of workers that may come into contact with these exposures because of the nature of their jobs, tasks, work schedules, or work environment. Identifying the subset of relevant potential exposures in the workplace can be accomplished by walkthrough surveys or audits, review of product safety data sheets or labels, past or current exposure monitoring, and literature reviews of known or suspected exposures in similar industries or occupations. On the other hand, observed symptoms or illnesses in a workplace, especially in situations where multiple exposures are known to be present, should prompt consideration of a CRA approach using an effects (outcomes)-based approach [49]. Under this approach, the first step is to identify the health effects of interest and potential concern and the specific group of workers experiencing these potentially related effects. Health effects may be identified based on worker complaints or the findings of workplace physicals, health surveys, medical evaluations, or epidemiologic literature of similar worker group exposures. Both approaches will require an understanding of which exposures may result in a combined health risk and their combined exposure and toxicity profiles. 

## 3. Occupationally Based CRAs in Practice (Three Examples)

We present three hypothetical examples below to illustrate how a simplified CRA approach can be applied in occupational settings today. The examples begin with scenarios and methods that OHS professionals are most familiar with pertaining to multiple chemical exposures, followed by less familiar and more complex concepts involving co-exposure to chemical and non-chemical agents and psychosocial stressors. Although the first example may be familiar to many in the field, it remains underutilized in many settings and is important to reiterate here. It also demonstrates how simple adaptations from this long-standing approach can be used to address more novel scenarios. 

### 3.1. Central Nervous System (CNS) Depression from Co-Exposure to Multiple Chemicals 

The most common method used worldwide by OHS professionals to evaluate cumulative risks is the OEL mixtures approach in which co-exposure to two or more chemicals that have a similar toxicological effect (i.e., act upon the same organ or system) are independently compared to their corresponding OELs; these ratios then are summed to assess the combined hazard [23,50,51,52,53]. Specifically, the threshold limit value^®^ (TLV^®^) mixture formula is as follows:

(1) $$TLV^{®}\;Mixture\;=\;\frac{C_{1}}{T_{1}}+\frac{C_{2}}{T_{2}}+\frac{C_{3}}{T_{3}}+\ldots \frac{C_{n}}{T_{n}}$$

*C_n_*–Measured chemical exposure
*T_n_*–*TLV*^®^ of the respective chemical

Although not an absolute bright-line, calculated values < 1 are generally not expected to pose a health risk to most workers, while values > 1 are considered more likely to pose a health risk. Accounting for multiple chemicals that yield the same toxicological effect is important because although individual chemical exposures may not be of concern (e.g., C_1_/T_1_ is < 1 and C_2_/T_2_ is < 1), combined chemical exposures may be problematic (e.g., C_1_/T_1_ + C_2_/T_2_ is > 1). The mixture formula is an example of dose addition. Note that this method is equivalent to the Hazard Index (HI) approach utilized by the USA Environmental Protection Agency [54] for evaluating non-cancer chemical hazards. 

As an example, consider the use of solvents and degreasers such as methyl ethyl ketone (MEK) and xylene in the metalworking industry [55,56]. These chemicals share a common critical health effect (i.e., CNS depression) via the same mode of action (i.e., the chemicals cause the same toxic effect by essentially the same sequence of major biochemical events in the body). Assume that a routine industrial hygiene monitoring survey of a metal fabrication shop reveals that a subset of workers are concurrently exposed to an 8-hr time-weighted average (TWA) airborne concentration of 108 parts per million (ppm) MEK (TLV^®^ = 200 ppm) and 64 ppm xylene (TLV^®^ = 100 ppm) during parts cleaning operations. If viewed independently, these two chemical exposures do not appear to be of concern (i.e., 108/200 = 0.54 and 64/100 = 0.64). However, the combined exposure in this decision index yields a value that exceeds unity (i.e., 1.18), thereby representing a scenario where workers’ co-exposure to two different, but toxicologically similar, chemicals may be of health concern. For this example, the risk management action is clear based on established regulatory requirements and recommended guidelines. That is, both the Occupational Safety and Health Administration (OSHA) [52] and the American Conference of Governmental Industrial Hygienists (ACGIH^®^) [23] recognize that chemical mixtures with values > 1 are indicative of potential health risk and one or more of the identified chemical exposures must be reduced. 

Under this scenario, some of the same shop workers who are exposed to MEK and xylene occupationally may experience similar types of chemical exposures outside the fence line. For example, hobbyist painters may use MEK or xylene as a thinner or solvent [55], whereas hobbyist boat builders may use a styrene resin (which is also associated with CNS effects) to construct fiberglass or Kevlar boats [57]. Certain personal risk factors may also modify the effects of CNS depression, such as excessive alcohol consumption [56]. Although many OHS professionals may be familiar with the TLV mixture approach, such non-occupational and personal risk factors are not usually considered in occupational health assessments. However, from a general public health perspective, focusing only on the workplace may be insufficient to protect or improve the desired health outcomes of the shop worker. Providing training and educational materials about the impact of outside the fence line exposures/personal risk factors is therefore suggested in those situations where such risk factors are known or suspected. 

### 3.2. Hearing Loss from Co-Exposure to Chemicals and Physical Agents 

Different types of chemical and non-chemical exposures in the workplace can result in the same health effect, and the combined risk from multiple exposures can exceed those predicted for the same exposures under an assumption of dose addition or response addition [23,58]. Two well-known examples relate to the combined lung cancer risk from asbestos and cigarette smoke [59,60,61,62,63,64] and risk of tuberculosis from silica-exposed workers with silicosis [65]. 

There is also growing evidence in occupational settings of the risk of hearing loss from co-exposure to noise (physical agent) and certain chemicals that are toxic to the neurological structures in the ear (i.e., ototoxicants). A number of studies have found that simultaneous exposure to moderate noise levels and organic solvents (e.g., carbon disulfide, hydrogen cyanide, toluene, styrene, trichloroethylene, xylene), asphyxiants (e.g., carbon monoxide), metals (e.g., lead), and pharmaceuticals (e.g., cisplatin, furosemide, salicylates, streptomycin) can damage the cochlea and lead to hearing loss [23,66,67,68,69,70,71,72,73,74,75,76]. These effects can occur via the same or different modes of action and the combined risk often is greater than would be predicted under an assumption of response addition [76]. For example, in rodent studies, exposure to noise alone has been found to injure the stereocilia, whereas exposure to toluene alone has been found to injure the outer hair cells. Even though the specific cells damaged by noise and toluene are different, the combined effect of these exposures has been found to cause a greater loss of hearing than would be expected from assuming response addition for each exposure [77]. To raise further awareness of this issue, ACGIH [78] recently added a qualitative “ototoxicant notation” (similar to the “skin notation”) as part of the TLV^®^ documentation. Consideration of both noise and ototoxicant exposures in the workplace is especially important because millions of dollars are spent each year in the United States on workers’ compensation for hearing loss disability [76], and even if a company has an effective hearing conservation program, hearing losses can still occur if concurrent exposures to ototoxicants are not addressed. 

Although OHS professionals routinely assess chemical and non-chemical exposures independently in the workplace, it is far less common to consider the combined effect from these exposures. There are likely two main reasons why this is the case. First, OHS professionals may not be aware of the combination of exposures that can lead to a particular health effect and limited guidance and tools are available for identifying such co-exposures in the field. Second, OELs are typically based on a single exposure with a specific unit of measurement, and there is no standard approach (such as the TLV^®^ mixture approach) for how to combine disparate exposures. Nonetheless, cumulative risks from many chemical and non-chemical exposures can be considered qualitatively or semi-quantitatively, and risk management actions can be taken in situations where co-exposures are suspected of posing a significant combined health risk. 

As an example, consider a machine shop where large sheets of metal are cut, welded, and fabricated to construct various equipment parts, and toluene is used daily to clean metal surfaces. Assume that annual audiograms recently conducted at the facility have demonstrated a loss in auditory function among long-time workers in the machine shop. Industrial hygiene surveys show that the 8-hr TWA noise levels in the machine shop have historically ranged from 80–83 decibels on the A scale (dBA) (TLV^®^ = 85 dBA), while the average 8-hr TWA air concentrations of toluene have ranged from about 10–15 ppm (TLV^®^ = 20 ppm). While the measured noise and solvent exposure levels do not appear to pose a health concern when evaluated independently, the observed hearing loss in this population could potentially be attributed to the combined risk from chemical and physical exposures. However, because the combined dose–response relationship for hearing loss attributable to both noise and toluene exposures is not known at this time, it is not possible to draw any definitive conclusions about health risk or to identify specific exposure reduction target levels. Despite such uncertainties, OSHA/NIOSH [76] has recently recommended that precautions be taken to reduce noise and chemical exposures when ototoxicants are identified in the workplace, including engineering and administrative controls and the use of personal protective equipment. In this example, one approach would be to voluntarily reduce the noise TLV^®^ to a facility-specific target level (i.e., action level) of 80 dBA and/or reduce the toluene TLV^®^ by ½ (i.e., facility-specific target level of 10 ppm) to account for potential combined effects from these co-exposures. It is not possible to utilize a TLV^®^ mixtures approach for this exposure combination because of differences in exposure units (mg/m^3^ vs. dBA), measurement scale (linear vs. logarithmic), and potential toxicological interactions (additivity vs. synergy). However, as a screening tool, a hazard quotient could be computed for noise and ototoxicant exposures independently and added together under an assumption of dose additivity. 

As above, it is important to recognize implications beyond the control of the employer or workplace in that some of the same shop workers who are exposed to noise and solvents occupationally may experience similar types of exposures outside the fence line. For example, a hobbyist race car driver, drummer, or user of ear buds may experience significant noise exposures, whereas a hobbyist painter may use toluene or another ototoxicant as a solvent or degreaser [79]. Selected personal risk factors, such as age, may also modify the effect of hearing loss in this population [80]. If known or suspected, providing training and education materials about outside the fence line exposures/personal risk factors and/or adopting better controls in the workplace is recommended. 

### 3.3. Cardiovascular Disease from Co-Exposure to Chemicals and Psychosocial Stressors

There is emerging scientific evidence that chronic psychosocial stress may make individuals more susceptible to health effects from physical and chemical exposures [81,82,83]. This greater susceptibility is believed to operate, in part, via allostatic load pathways of stress-related impacts on immune, endocrine, and metabolic function [84]. As a result, workplace physical and chemical exposures may have disproportionately greater impacts among highly or chronically stressed workers. 

One example of this relationship is the combined effect of co-exposure to chronic stress and heavy metals (e.g., lead [Pb]) on cardiovascular disease (CVD), blood pressure [85], and neurocognitive outcomes [86,87,88]. In occupational settings, studies have documented associations between work-related psychosocial stressors (e.g., job strain, job grade, job status, unemployment, conflict with management, workplace culture) with hypertension and CVD [43,89,90,91,92,93,94]; with some noted gender differences [95]. Substantial evidence has also shown associations between Pb and CVD, even at relatively low exposure levels [96]. Furthermore, animal studies have revealed that exposures to chronic stressors (e.g., restraint or foot-shock) can heighten the impacts of controlled Pb doses [86,97,98], while epidemiologic studies have found heightened impacts of Pb on blood pressure [85] and cognitive outcomes [88] with chronic stress. Taken together, these multiple lines of evidence suggest the responses to combinations of psychosocial stressors and metal exposures could be additive, or possibly greater than additive.

Most OHS professionals are familiar with features of the work environment that may cause stress or discomfort (e.g., heat, noise), although these exposures are generally assessed as physical entities (i.e., using physical measures of temperature or sound pressure), rather than as chronic stressors (i.e., sources of perceived disruption, annoyance, or interference with concentration). This under-emphasis on psychosocial stressors or on stress-related aspects of physical exposures may be due to limited familiarity with the impacts of chronic stress on health or a lack of knowledge or oversight for measuring, interpreting, and intervening on psychosocial stressors. Overcoming these latter issues will likely require collaboration with workplace psychologists, audiologists, and other professionals. 

To date, no OELs have been developed for psychosocial stressors in the workplace, with the exception of some common non-chemical exposures operating through multiple pathways (e.g., heat, noise). However, in these cases, OELs are based on the physical properties of these stressors (i.e., sound pressure in A-weighted decibels) rather than on psychosocial impacts (i.e., perceived disruption, annoyance). A large body of occupational psychology literature has examined psychosocial stressors and developed appropriate scales for their quantification [99,100]. As above, the lack of OELs or standardized tools in OHS may be due, in part, to inherent challenges in measuring psychosocial stress. Psychosocial stressors that are critical to one population may also have no bearing on another population and impacts via stress-related pathways are highly dependent on individual appraisal (i.e., perception) of the stressor. For example, although many workers in a given location may be exposed to comparable levels of sound pressure, only a subset will find the “noise” to be bothersome or disruptive. 

Note that there is often a desire for simple, objective physical measures of stress (i.e., biomarkers). However, by definition, stress is a non-specific multi-systemic process that originates with an individual’s perception of annoyance or risk. Therefore, although many immune and inflammatory markers are often elevated under chronic stress, there is no definitive biomarker for stress exposure or effects. Even those biomarkers commonly used to assess acute stress (i.e., blood cortisol levels) present substantial challenges of interpretation, both individually and in interaction with chemical exposures. Notably, chronic stress can lead to reduced cortisol production and a flattened diurnal rhythm, further complicating interpretability [101]. 

Although more research is needed to fully incorporate psychosocial stressors into occupational risk assessments, the potential cumulative risk from chemical or non-chemical and psychosocial stressors can at least be considered qualitatively in many situations. For example, consider a group of front-line production workers in a fast-paced, high-demand manufacturing facility where Pb is used to construct or weld metal parts. Assume that the plant is undergoing major structural and managerial changes or that an anonymous survey (important in settings of perceived job insecurity) suggests that a substantial portion of workers are experiencing elevated perceived stress or symptoms potentially indicative of chronic stress (e.g., fatigue, moodiness, weight loss, sleep loss, increased injury rate). If monitoring data reveal average 8-hr TWA Pb exposures ranging from 0.01–0.04 mg/m^3^ (TLV^®^ = 0.05 mg/m^3^) for workers on the production line, although the measured Pb exposures by themselves do not exceed the health-based OEL, the known addition of a high-stress environment could be cause for concern. Despite lacking a definitive quantitation of the risk posed by this combination, a voluntary reduction (at least temporarily) in the lead TLV^®^ and/or changes in working conditions (e.g., more frequent breaks, more comfortable conditions) could potentially ameliorate the potential risk. 

Exposures to psychosocial stressors outside the fence line, which may be more prevalent among lower-income workers or those in less-developed countries [102], may impact susceptibility to physical or chemical exposures at work. That is, chronic stressors in the community or home environment (e.g., high crime neighborhoods, family strain) may contribute to allostatic load [103], thereby shaping workers’ susceptibility to physical exposures in all aspects of their lives. Workers may also encounter chemical or physical exposures outside the fence line that can combine with psychosocial stressors from the workplace. For example, many older, lower-income residences have historical deposition of Pb from paints or vehicular exhaust near major roadways [104,105], and increasingly, communities near e-waste and scrap metal recycling facilities struggle with chronic metals exposure [106]. In sum, some workers (particularly lower-income manufacturing or industrial workers) may be more highly *exposed* to metals in the workplace and also more susceptible to those exposures due to chronic psychosocial stressors. 

## 4. Implications for Occupationally Based CRA Research and Future Practices

There are an estimated 2.78 million occupationally related deaths globally per year with 86% attributed to work-related diseases and 14% due to fatal accidents [107]. This represents 5–7% of total deaths worldwide [107,108,109,110]. Further, it has been estimated that globally 430 million persons per year suffer serious non-fatal injuries or illnesses from work-related causes [109]. The total cost of these accidents, illnesses, and deaths account for 4% of the world gross national product [109]. Although definitive data are lacking, much of this disease burden may be driven by combinations of chemical and non-chemical exposures, perhaps coupled with modifying personal risk factors. Implementing a CRA approach for occupationally based health risk assessments that considers co-exposures and possible toxicological interactions could therefore increase work productivity and improve worker health and safety in a tangible and significant way. 

A comprehensive CRA framework that integrates chemical and non-chemical exposures from occupational and non-occupational domains with personal risk factors has yet to be developed or implemented in practice [4,5]. However, OHS practitioners can begin taking a CRA perspective when evaluating multiple exposures and health effects in the work environment using the preliminary guidance and initial framework described above. In particular, a CRA approach that encourages consideration of co-exposures leading to the same health outcome can be incorporated into current job safety analyses, hazard assessments, and risk assessments. Voluntary risk management actions to control or limit potential or suspected co-exposures of concern can then proactively be adopted on a case-by-case basis. 

As discussed below, additional documentation or research in several areas, similar to that developed by OSHA/NIOSH [76] for noise and ototoxicant exposures, could also lead toward a practical approach for identifying relevant co-exposures and evaluating cumulative risks in the workplace. 

First, reliable (yet simple) guidance and tools are needed to identify relevant combinations of chemical and non-chemical exposures (including psychosocial stress) that are associated with various industries or processes. Although standardized check-lists, walk through surveys, or job hazard analyses (JHAs) are common among OHS professionals, these tools are typically used to identify general workplace exposures (e.g., chemical release) or physical hazards (e.g., noise) rather than concurrent exposures that might occur during certain job processes or tasks that can lead to the same health outcome. These tools could readily be modified to account for such co-exposures and gather information on mode of action information for identified exposures. An example checklist concept that addresses these needs has been previously presented [111]. Additionally, existing tools generally do not account for personal risk factors that may modify the combined health effect, but these tools could be modified to consider such attributes. Note that several existing tools developed in other contexts may also provide a useful starting point for conducting occupationally based CRAs (see Table 3).

Second, OEL development based on the combined risk from chemical and non-chemical exposures in the workplace is an area in need of future research. The chemical mixture TLV^®^ is a common approach that has been used for decades to assess the combined (additive) risk from multiple chemical exposures with the same health effect, but such applications do not account for non-chemical exposures, modifying factors, or potential interactions. As mentioned, ACGIH [78] has announced that it will add an ototoxicant notation for relevant chemicals, which will provide a useful qualitative indicator of when to consider co-exposure to these chemicals and noise in occupational settings. While this notation only provides a qualitative indicator, and only one chemical to date (i.e., styrene) has an established TLV^®^ for which hearing loss is identified as one of the critical effects, ACGIH is clearly moving CRA concepts into their TLV process. However, more work is needed to determine how OSHA, NIOSH, ACGIH, and other organizations can better incorporate CRA approaches within existing or new frameworks for use among OHS professionals. ATSDR [38] has also developed a framework for assessing toxicological interactions from multiple chemical exposures commonly encountered at contaminated sites that may be adaptable to OEL development. It is important to recognize that although reducing exposures to protect against the most critical effects may protect against other effects associated with combined exposures, not all OELs are based on the most critical health effect. These critical health effects may also be informed by further research into understanding the roles of biomarkers of effects for combined exposures. Protecting the worker therefore requires an understanding of how the critical health effect is impacted by both chemical and non-chemical exposures, which may also be modified by personal risk factors. 

In the interim, OHS professionals can take steps to set voluntary (site-specific) action limits or target values below existing OELs to reduce the risk of adverse health effects from combined exposures [47]. As suggested in the examples above, this may take the form of voluntarily lowering existing chemical or non-chemical OELs (or both) on a site-specific or industry-specific level, depending on the measured exposure levels and what is known about the nature of the combined risk (e.g., co-exposures resulting in greater-than-additive effects may encourage adoption of a lower action level than those where the effects are additive). It is important to recognize that the magnitude of voluntary reductions is not a precise or pre-defined level based on known dose–response data and will require judgements and acceptance by the OHS practitioner, the organization’s management, and others on a site-by-site basis (e.g., process engineer, medical personnel, etc.). Risk management approaches, such as NIOSH’s process for occupational exposure banding, which is largely based on the Globally Harmonized System for Classification and Labelling of Chemicals (GHS) hazard codes, and NIOSH TWH guidelines may provide useful frameworks for establishing target exposure control limits or ranges to reduce risks among workers from combined exposures when there are limited toxicological data [113]. Greater adoption of a CRA approach can also improve existing tools available through the TWH program by helping to identify those co-exposures of greatest concern, and hence in greatest need of risk management action, in specific occupations or industry sectors. 

Third, the focus on potential cumulative risks in the workplace will necessitate the future development and dissemination of appropriate health education and risk communication materials to workers. Development of these materials will help fulfill OSHA’s Hazard Communication Standard (HCS) requirement that employers develop a hazard communication plan and provide training and education to employees about hazards in the workplace, including chemical mixtures [114,115]. The purpose of such materials is to ensure that health hazard information is shared with potentially exposed workers, including information on known or suspected co-exposures and personal risk factors, so that employees can be fully informed and have the ability to seek or take precautionary measures (some of which may occur outside the fence line). For example, when asbestos and smoking exposures were found to result in a combined (greater than additive) lung cancer risk in the early 1970s, employees who routinely worked with asbestos materials (e.g., insulators) were advised by academic researchers and government agencies to discontinue smoking as well as use appropriate control measures as a means of reducing their asbestos-related disease risks [59]. Because an in-depth understanding of many non-chemical exposures requires substantial expertise, greater collaboration in the future between OHS professionals and the wider occupational health community (e.g., workplace audiologists, psychologists, behaviorists, and others) will be essential.

It is important to recognize that although an occupationally based CRA may include consideration of exposures occurring outside the fence line or personal risk factors that can modify a health outcome, the intent of this approach is not to exonerate an employer from its responsibility to provide a “place of employment which is free from recognized hazards that are causing or are likely to cause death or serious physical harm…” as described by the OSH Act [116]. The CRA approach presented here focuses initially (or solely) on exposures that occur within the fence line, with possible consideration of personal risk factors or exposures occurring outside the fence line only if it is anticipated that these additional efforts would potentially improve worker health. 

## 5. Conclusions

In this perspectives article, we further introduce OHS professionals to the CRA approach and offer preliminary guidance on when it might be important to conduct an occupationally based CRA in the workplace and how to set realistic boundaries. Although we identify several areas that pose scientific or social challenges, we recommend simple (voluntary) risk management and risk communication solutions that can be taken in the near-term. Adopting a CRA perspective can lead to improved worker health and safety, as well as improvements in other related areas such as the environment and general public health and product stewardship and sustainability. This approach complements other health-based programs and initiatives designed to take a more holistic view of the various factors that contribute to worker health (e.g., NIOSH’s TWH program). We recognize that the field is evolving and new tools and approaches and a more comprehensive CRA framework are likely to be available in the future. 

## Figures and Tables

**Table 1:** Common terms used to describe cumulative risks.

Term	Definition and Rationale
Additivity	When the “effect” of the combination is estimated by the sum of the exposure levels or the effects of the individual chemicals. The terms “effect” and “sum” must be explicitly defined. Effect may refer to the measured response or the incidence of adversely affected animals. The sum may be a weighted sum (see “dose addition”) or a conditional sum (see “response addition”) [14].
Aggregate Exposure	Exposure to same stressor from all sources and multiple exposure routes (e.g., inhalation, oral, dermal) [5,15].
Antagonism	When the effect of the combination is less than that predicted by the component toxic effects. Antagonism must be defined in the context of the definition of “no interaction,” which is usually dose or response addition [14].
Cumulative exposure	Aggregate exposure from all sources to multiple entities, including chemical, physical, and biological agents as well as psychosocial stressors, that affect the same or different health effects. It also can include the absence of a necessity. The USA Environmental Protection Agency (EPA) [15] describes cumulative exposure more narrowly as “total exposure to multiple agents *that cause a common toxic effect(s) on human health by the same, or similar, sequence of major biochemical events*” (emphasis added). It can also include a syndrome of effects. Clearly the scope of an assessment involving cumulative exposure will affect the breadth of entities and stressors considered [8]
Dose addition	When each chemical behaves as a concentration or dilution of every other chemical in the mixture. The response of the combination is the response expected from the equivalent dose of an index chemical. The equivalent dose is the sum of component doses scaled by their toxic potency relative to the index chemical [14]. Method typically applied when two or more chemicals share a similar mode of action or affect the same target organ. Sometimes referred to as “simple similar action.”
Interaction	Howard and Webster [16] observe that “epidemiologists and toxicologists approach interaction assessment by defining a noninteractive model; departures from the model are then considered interactive”. In toxicology, this term describes a toxicological response of two or more chemicals that differs from the predicted (additive) response (i.e., dose addition or response addition is the null hypothesis). Can occur during pharmacokinetic or pharmacodynamic processes [3,17]. In epidemiology, the use of this term is more complicated compared to its use in chemical mixtures toxicology, in part, because the term, “interaction” describes different phenomena [18]. Analyses of biological interactions examine two or more causes and the influence of the combination of causes on biological responses (e.g., a disease). Statistical interaction describes the presence of variables that may be interacting on the effect measures being examined; thus, would necessitate an interaction term in a statistical model of multiple exposures to improve model fit [16,18,19]. While these relatively simple descriptions are provided to convey a general understanding, the referenced manuscripts and references therein should be consulted for in-depth treatments of this topic.
Mixed Exposure	Exposures to either chemical mixtures, different substances at different times, simultaneous exposure to multiple substances, or simultaneous exposure to a chemical substance and another stressor [20].
Mode of action	Describes a biologically plausible series of key events leading to an effect [21]. An effect typically refers to a functional or anatomical change, at the cellular level, resulting from an exposure.
Modifying factor	A factor that differentially (positively and negatively) modifies the observed effect of a risk factor on disease status. Effect modification occurs when the magnitude of the effect of the primary exposure on an outcome (i.e., the association) differs depending on the level of a third variable. This is often contrasted with confounding that occurs when the effect or association between an exposure and outcome is distorted by the presence of another variable [19].
Response addition	When the toxic response (rate, incidence, risk, or probability of effects) from the combination is equal to the conditional sum of component responses, as defined by the formula for the sum of independent event probabilities. For two chemical mixtures, the body’s response to the first chemical is the same whether or not the second chemical is present [14]. Method applied when two or more chemicals cause a common effect through different (independent) toxic mechanisms. Applies when chemicals are toxicologically dissimilar (follows the probability law of independent events). Sometimes referred to as “simple independent action.”
Risk	The Society of Risk Analysis describes “risk” in relation to “the consequences (effects, implications) of this activity with respect to something that humans value [22]. The consequences are often seen in relation to some reference values (planned values, objectives, etc.), and the focus is often on negative, undesirable consequences. There is always at least one outcome that is considered as negative or undesirable.”
Stressor	Any physical (e.g., sunlight, heat, cold), chemical, or biological (e.g., viruses, bacteria, fungi) entity that can induce an adverse response. A stressor may also be the lack of an essential entity. The stressor may not cause harm directly, but it may make the target more vulnerable to harm by other stressors [8]; the term’s use has also included psychosocial (e.g., community violence, community crime, non-voluntary unemployment) entities [9]. Although the term “stressor” has often been used to refer to any chemical or non-chemical agent that can have an effect on the biological functioning of an organism, in this paper we distinguish between agents that operate through physical pathways (e.g., chemical, biological, or physical exposures) and those operating through psychosocial pathways (e.g., perceived stress). We therefore use the term “exposure” to refer to any chemical or non-chemical agent (including psychosocial stressors) and reserve the use of the term “stressor” when referring only to psychosocial stressors.
Susceptibility	Refers to the condition of differential or heightened responses in a population relative to another population. Some individuals in the workplace (or general population) may be more susceptible to the effects of an exposure due to differences in genetic and epigenetic predisposition, health status (e.g., immune-compromised conditions), lifestyle factors (diet, obesity, smoking status, alcohol abuse), age, ethnicity, sex, medications, and other factors [23,24,25].
Synergism	When the effect of the combination is greater than that suggested by the component toxic effects. Synergism must be defined in the context of the definition of “no interaction,” which is usually dose or response addition [14].
Vulnerability	Refers to the condition of differential or heightened exposures relative to those experienced by another population. This can include differences in historical exposure, body burden, and other sources of exposure. Vulnerability can vary within and between workplaces as well as across the general environment. In this manuscript, the term “vulnerability” is used more narrowly; for example, Kasperson [26] includes differential exposure as one of four categories of vulnerability. See also EPA [8].

**Table 2:** Examples of chemical and non-chemical stressors and personal risk factors encountered in occupational and non-occupational domains.

Domain	Chemical and Non-Chemical Stressors			
	Chemical	Biological	Physical	Psychosocial
Occupational (within fence line or employer control)	Metals	Bacteria	Noise	Noise
	Volatile organic compounds (VOCs)	Virus	Heat/Cold	Heat/Cold
	Particulates	Mold	Radiation (ionizing/non-ionizing)	Job strain
	Aerosols	Endotoxins	Ergonomics	Job grade
	Pesticides	Bloodborne pathogens	Physical exertion	Shiftwork
		Allergens	Shiftwork	
Non-Occupational (outside fence line or employer control)	Metals	Bacteria	Noise	Noise
	Volatile organic compounds (VOCs)	Virus	Heat/Cold	Heat/Cold
	Particulates	Mold	Radon	Poverty
	Aerosols	Endotoxins	Radiation (ionizing/non-ionizing)	High-crime neighborhood
	Pesticides	Bloodborne pathogens	Ergonomics	
		Allergens		
Personal Risk Factors (apply to both occupational and non-occupational environments)	Age, Sex, Health Status, Obesity, Smoking, Alcohol Consumption, Drug Use			

**Table 3:** Existing tools that could assist in conducting occupationally based CRAs.

CRA Tool	Description
Mixtures of substances in the workplace (MIXIE)	http://www.irsst.qc.ca/mixie/?en
	An exposure-based approach online tool designed at the University of Montreal that evaluates possible additive or interactive effects of chemical in the workplace. The underlying toxicological database includes 695 chemicals [112]. Users input chemical air monitoring data for one or more chemicals, which are evaluated for common toxicological endpoints and potential health risks. For example, if the chemicals toluene (S1), ethyl benzene (S2), and acetaldehyde (S3) are identified, MIXIE highlights 2 classes of toxic effects for these chemicals (i.e., eye involvement and upper respiratory tract involvement) and 3 additional classes of toxic effects for S1 and S2 (i.e., CNS involvement, auditory system involvement, and embryonic or fetal disorders). S2 and S3 are also identified as recognized carcinogenic substances by some organizations. This tool does not include non-chemical exposures or personal risk factors and presupposes that chemical co-exposures have already been identified by the OHS professional (i.e., it does not provide guidance a priori on which co-exposures may be important to evaluate for a particular industry or work processes). This tool also generates mixture-based OELs that may be based on different critical effects, but the default OELs for the individual chemicals provided within MIXIE are not adjustable.
Haz-Map	http://hazmap.nlm.nih.gov
	An effects-based approach online tool to identify exposures associated with occupational or non-occupational activities. The user can select from several categories of exposures including “occupational diseases”, “high risk jobs”, “industries”, “job tasks”, “processes”, “symptoms”, or “non-occupational activities”. Numerous selections are available in each of these categories which provide outputs of specific chemical, biological, and physical agents related to the selected exposures.
Wireless Information System for Emergency Responders (WISER)	https://wiser.nlm.nih.gov/
	An online tool developed to assist emergency responders in hazardous material incidents. As part of this tool, users can input one or more observed health effects, which are used to generate a list of potential chemical exposures associated with these effects. For example, if the health effects eye irritation/redness and chest pain are observed, WISER identifies 199 possible chemical exposures. The chemicals identified could then be further limited by comparison to the chemical inventory in the workplace. However, as above, this tool is only focused on chemical exposures and does not identify possible exposure combinations that may interact or personal risk factors that may modify the health effect.
Online interactive Risk Assessment (OiRA)	https://oiraproject.eu/en/
	An online tool maintained by the European Agency for Safety and Health at Work (EU-OSHA) and developed to assist micro and small enterprises in conducting standardized risk assessments for different sectors or types of exposures (e.g., agricultural, petrol stations, pharmacies, psychosocial risks, physical agents). For example, this tool explores general health and safety issues associated with the leather and tanning industries and asks a series of questions on several topics including chemical use, use of machinery and tools, office work, and organization work factors. At the end of the process, it provides a decision-making and risk management tool to allow the user to prioritize and address the workplace risks identified. This tool does not appear to explicitly address the risks of combined exposure, but it may help OHS professionals categorize a broad range of risks that could be more easily evaluated with a cumulative risk approach.

## Author Statements

The findings and conclusions in this report are those of the authors and do not necessarily represent the official position of the National Institute for Occupational Safety and Health, Centers for Disease Control and Prevention. The views expressed in this article are those of the authors and do not necessarily reflect the views or policies of the USA EPA. Mention of trade names or commercial products does not constitute endorsement or recommendation of use. Links to non-USA EPA websites do not imply any official USA EPA endorsement of or a responsibility for the opinions, ideas, data, or products presented at those locations or guarantee the validity of the information provided.
